# Overcoming the Dexter distance constraint: a relay strategy for asymmetric photocatalysis

**DOI:** 10.1093/nsr/nwag410

**Published:** 2026-07-06

**Authors:** Hao Zeng, Jie Wu

**Affiliations:** Department of Chemistry, National University of Singapore, Singapore; Department of Chemistry, National University of Singapore, Singapore

**Figure 1.**
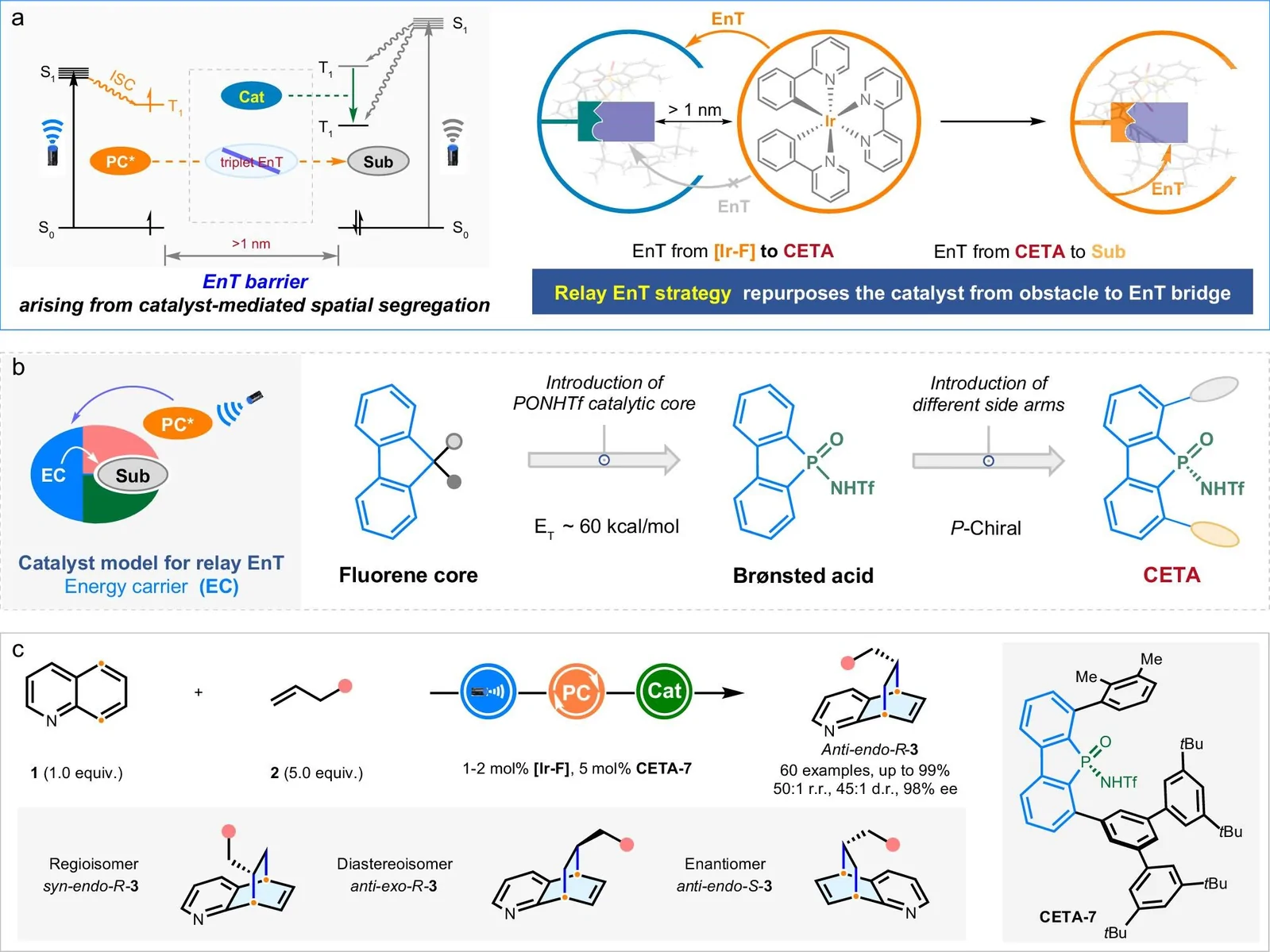
(a) EnT barrier imposed by Dexter distance constraints in a spatially segregated dual-catalytic system. (b) Catalyst design integrating an energy carrier, a Brønsted acid activation site and chiral recognition elements. (c) CETA-7-enabled asymmetric dearomatic [4 + 2] cycloaddition of quinolines with alkenes. [Ir**-**F], Ir[dF(CF_3_)ppy]_2_(dtbbpy)PF_6_; Cat, catalyst; PC, photocatalyst; S, singlet; Sub, substrate; T, triplet. Reproduced from Figs 1 and 2 of Ref. [6] with permission.

Photocatalysis has reshaped synthetic chemistry by harnessing light to drive transformations inaccessible in the ground state. While single-electron transfer pathways have matured into a robust platform [1,2], triplet energy transfer (EnT) represents a complementary mode with vast, yet underexplored, potential—particularly in asymmetric synthesis [3,4]. In conventional asymmetric EnT catalysis, a chiral catalyst is often introduced to bind the substrate, lower its triplet energy and induce stereocontrol. However, triplet-energy matching alone is insufficient. According to Dexter theory, efficient EnT requires close spatial proximity and orbital overlap between the donor and acceptor, with the transfer rate decaying exponentially as distance increases [5]. Consequently, the chiral environment necessary to organize a substrate inherently segregates it from the photosensitizer. This creates a hidden, yet profound, EnT barrier that has largely been overlooked (Fig. 1a).

In a recent paper published in *Science*, Tan and co-workers identified this spatial constraint as a fundamental limitation in asymmetric EnT photocatalysis and ingeniously turned it into a catalyst-design principle [6]. To overcome the Dexter distance constraint, they proposed a relay EnT paradigm in which the chiral catalyst is no longer a passive stereochemical director or a steric obstacle, but actively serves as an EnT mediator. Guided by this concept, the team engineered a novel class of chiral energy transfer acids (CETAs) (Fig. 1b). The brilliance of the CETA design lies in its modular architecture, which seamlessly integrates three components: a high-triplet-energy fluorene-like energy carrier, an N-triflyl phosphinamide Brønsted acid catalytic site and highly tunable chiral side arms. This design cleanly separates visible-light absorption (handled by an iridium photosensitizer) from stereochemical control (handled by the organocatalyst), while strategically positioning the energy carrier close to the bound substrate to bridge the physical gap.

To validate their strategy, the authors revisited the dearomative [4 + 2] cycloaddition of quinolines with alkenes, a reaction previously reliant on stoichiometric acidic activators and lacking asymmetric control [7]. Conventional chiral Brønsted acids gave poor activity, and 1,1'-2-bi-naphthol (BINOL)-based acids could even inhibit the reaction, consistent with EnT competition and spatial blocking. In contrast, the optimized CETA-7 exhibited remarkable activity. As shown in Fig. 1c, utilizing merely 5 mol% of CETA, the visible-light-driven cycloaddition proceeded with exceptional efficiency and outstanding control over regio-, diastereo- and enantioselectivity. The reaction demonstrated broad tolerance across >60 examples, including the successful late-stage diversification of complex, drug-derived substrates.

Extensive mechanistic studies, including Stern–Volmer analysis and density functional theory, confirmed that CETA effectively circumvents the EnT barrier. Most decisively, nanosecond transient absorption spectroscopy provided direct observation of the sequential energy flow: from the excited iridium complex, to the CETA catalyst and finally to the quinolinium substrate. Furthermore, the researchers uncovered a highly beneficial secondary effect: CETA acts as a transient energy reservoir, significantly prolonging the excited-state lifetime of the reactive triplet quinolinium from roughly 2 ns to over 80 ns, further underpinning the system’s exceptional catalytic efficiency.

This work fundamentally reframes asymmetric EnT catalysis from a purely energy-matching problem into a spatially resolved energy-delivery problem. By successfully embedding an adjustable energy carrier into a chiral acid catalyst, Tan and co-workers have provided a general and actionable blueprint for integrating substrate activation, stereochemical induction and excited-state energy logistics. This relay EnT concept promises to inspire a new generation of catalyst designs, unlocking transformations where direct EnT is currently curtailed by distance, steric shielding or incompatible chromophores, thereby significantly expanding the boundaries of asymmetric photocatalysis.
